# Phototherapy and Tailored Brushing Method. Personalized Oral Care in Patients with Facial and Dental Trauma. A Report of a Case

**DOI:** 10.3390/healthcare9050561

**Published:** 2021-05-11

**Authors:** Gianna Maria Nardi, Fabrizio Guerra, Artnora Ndokaj, Denise Corridore, Marsha Alicia Straker, Pasquale Sportelli, Roberto Di Giorgio, Felice Roberto Grassi, Roberta Grassi, Livia Ottolenghi

**Affiliations:** 1 Department of Oral and Maxillo-Facial Sciences, Sapienza University of Rome, Via Caserta 6, 00161 Rome, Italy; giannamaria.nardi@uniroma1.it (G.M.N.); fabrizio.guerra@uniroma1.it (F.G.); denise.corridore@uniroma1.it (D.C.); marshastraker@yahoo.com (M.A.S.); roberto.digiorgio@uniroma1.it (R.D.G.); livia.ottolenghi@uniroma1.it (L.O.); 2 Complex Unit of Odontostomatology, 70122 Policlinico Bari, Italy; info@sportellistudio.it; 3 Department of Basic Medical Sciences, Neurosciences and Sense Organs, University of Bari Aldo Moro, 70122 Bari, Italy; feliceroberto.grassi@uniba.it; 4 Department of Biomedical Sciences, University of Sassari, 07100 Sassari, Italy; grassi.roberta93@gmail.com

**Keywords:** phototherapy, personalized oral care, at-home oral hygiene, dental trauma

## Abstract

(1) Background: Traumatic dental injuries are frequent in children and young adults. The facial structures involved in dental trauma may include soft tissues of the face and mouth, bone and dental structures. Dental trauma often results in augmented dental anxiety. Phototherapy can improve stress and pain control thereby improving compliance in young patients with the necessary dental treatments, after dental trauma has occurred. (2) Methods: Phototherapy was performed to enable soft tissue healing. The Tailored Brushing Method (TBM), a personalized approach for at-home oral hygiene procedures, was also utilized, with the aim of improving biofilm control in traumatized patients. (3) Results: The approach hereafter presented made it possible to obtain subjective control of anxiety and pain documented on a visual analog scale (VAS) due to the innovative use of photo-biomodulation. In addition, for the first time, the TBM was adapted to the needs of a patient with facial trauma and illustrated. (4) Conclusions: Phototherapy and TBM were found to be effective in the combined treatment of soft tissue wounds and in the oral care of the traumatized patient.

## 1. Introduction

Traumatic dental injuries are frequent in children and young adults, accounting for 5% of all injuries. Twenty-five percent of all school children experience dental trauma and 33% of young adults have experienced trauma to the permanent dentition [1]. The literature shows that individuals with a dental trauma before the age of 6–8 years present a higher risk of experiencing another trauma in the late adolescent period. Moreover, individuals with augmented overjet, II class malocclusion, with oral breathing and augmented body mass index, present a higher risk of experiencing a dental trauma [2]. In addition, strong evidence exists that the level of dental anxiety is higher in pediatric patients with a history of dental trauma [3].

After a dental trauma occurs, both patient and caregivers are advised regarding overall behavior and special care of the injured oral area (tooth/teeth, soft tissues, alveolar bone) for optimal healing. Soft diet, avoidance of contact sports, use of a soft toothbrush, and meticulous oral hygiene and rinsing with an antibacterial agent, such as alcohol-free chlorhexidine gluconate 0.12% for 1–2 weeks, are the instructions delivered to the traumatized patient [1]. However, the adherence to the at-home instructions may be hampered by lack of patient compliance, caused by pain after injury, affecting both the tooth/teeth and soft tissues. This can be a particular concern in pediatric and adolescent patients [3].

Management of tooth injuries is a treatment that requires multidisciplinary cooperation of many specialists, which is required over a relatively long period of time [4,5,6]. As part of planning such a therapeutic procedure, which requires, inter alia, precise and timed observations, photobiomodulation can be included for use at various stages of the treatment of dental injuries. This approach can be used to treat pain immediately after trauma, and then to manage swelling and inflammation, and to speed up soft tissue healing [7,8]. However, the application of phototherapy in dental trauma has been described in few in vivo and in vitro studies. Phototherapy has been proven to increase angiogenesis in periodontal tissues of replanted teeth in rats [9,10]. Moreover, low-intensity photobiomodulation has been shown to be effective in enhancing the growth of stem cells taken from exfoliated deciduous teeth during situations of nutritional deficiency and avulsion [11].

Photobiomodulation is widely described in the literature. The effects of application of low-laser-light therapy (LLLT) on the tissues are: (i) acceleration of healing times; (ii) induction on neovascularization; (iii) pain control; and (iv) reduction of inflammation [12].

Bioptron polarized light has been shown to be effective in microcirculation improvement [13,14], periodontal tissue regeneration, decrease in inflammation [15,16], and pain and stress relief, without any side-effects. Moreover, Bioptron Hyperlight Therapy has been used to implement the protocols of non-surgical periodontal therapy. A recent clinical study showed a significant improvement in clinical scores and a better patient response to treatment (ref). Moreover, Bioptron is considered to be a non-aggressive, safe and cost-effective therapeutic option for the treatment of disorders of the mucosa and the musculoskeletal system, or as an appropriate treatment for skin eruptions to achieve skin regeneration and cicatrization [17,18].

The primary aim of this paper is to describe the phototherapy application and effects, both on soft and mineralized traumatized tissues, with positive documented outcomes on patients’ compliance. A secondary aim is to describe the at-home Tailored Brushing Method (TMB) for patients after dental trauma.

## 2. Case Presentation

### 2.1. Case Report

#### 2.1.1. Initial Situation

An 18-year-old Caucasian male patient, with ulcerative colitis, came to our attention, about 2 weeks after a drug-induced car accident. The patient was hospitalized for 7 days at the Plastic Surgery Department of the University Polyclinic in Bari, Italy. The patient presented with a nasal septum fracture, a bruise wound at the level of the upper lip, and several scrapes and bruises at the level of the mid-third of the face at the pre-maxillary level.

The study received the approval of the Ethical Committee (n.4743) and signed informed consent was obtained from the patient. All procedures were undertaken in accordance with the 1964 Helsinki Declaration and its later amendments or comparable ethical standards.

The PRICE 2020 checklist for case reports was used in this study.

Extra-oral and intra-oral photographic documentation was obtained using a digital camera (Nikon D7100, 105 mm Macro lens, R1C1 Macro flash) with standardized camera settings (shutter speed 1/80 and aperture settings f32 for intra-oral documentation; and shutter speed 1/100 and aperture settings f10 for extra-oral documentation).

**Figure 1:**
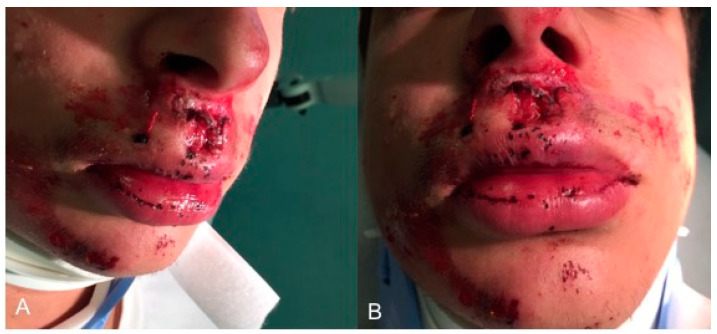
Extraoral facial images immediately after trauma: (**A**) extraoral lateral view; (**B**) extraoral frontal view.

The images of the face of the patient immediately after the trauma are represented in Figure 1.

**Figure 2:**
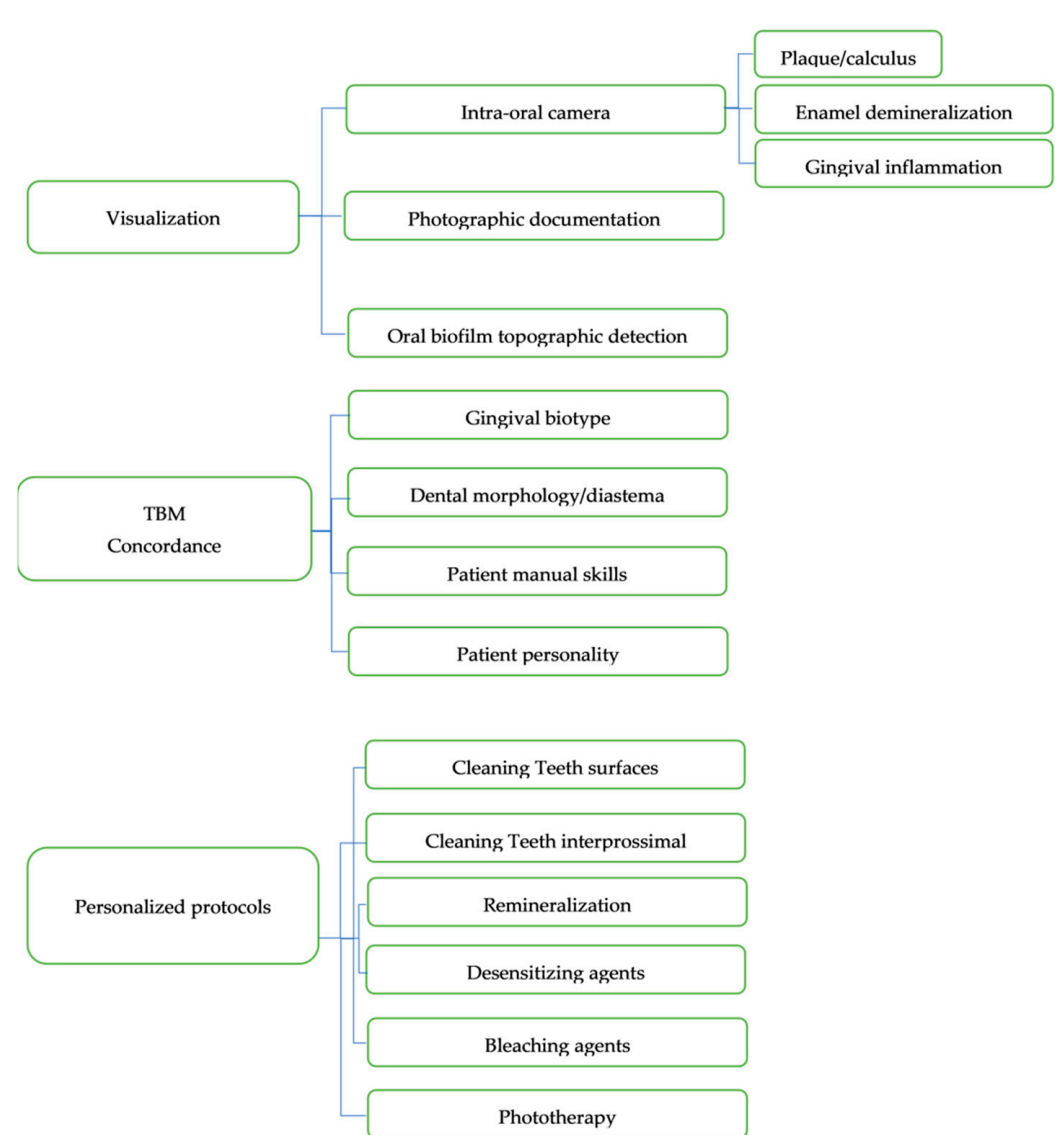
Flowchart with decision tree on personalized protocols in patients with facial/dental trauma based on TBM.

A decision tree summarizing the steps of the first stage of the TBM is shown in Figure 2.

#### 2.1.2. Initial Clinical Evaluation

##### Intraoral Camera Images

**Figure 3:**
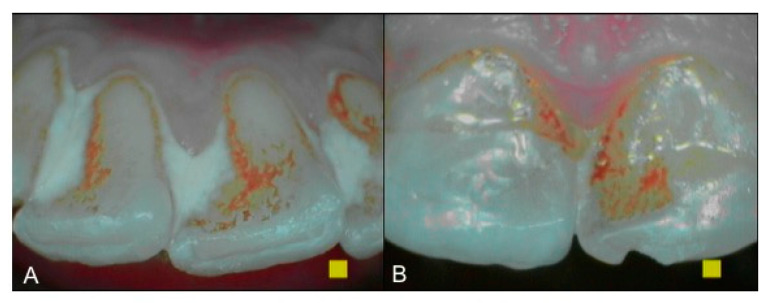
(**A**,**B**). Intraoral camera Soprocare images of calculus on lower and upper incisors.

**Figure 4:**
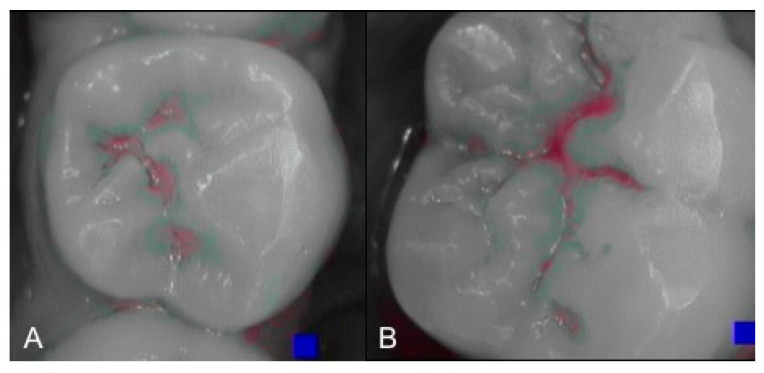
(**A**,**B**). Intraoral camera Soprocare images of active demineralization processes on the occlusal surface of the teeth.

An intraoral camera Soprocare (Acteon) was used to detect caries, plaque, calculus and gingival inflammation, one month after trauma (Figure 3 and Figure 4).

#### 2.1.3. Oral Biofilm Topography

The D-BIOTECH CLINICAL APPROACH is a topographic technique in dentistry used for biofilm detection.

Patient motivation occurs through presentation of intra-oral images showing bacterial biofilm residue to improve at-home oral hygiene, with particular care applied to the most plaque retentive sites, and to avoid inflammation and/or demineralization in these areas. This approach is aided by the selection of the most appropriate technologies, tools and clinical approaches for de-plaquing and debridement.

**Figure 5:**
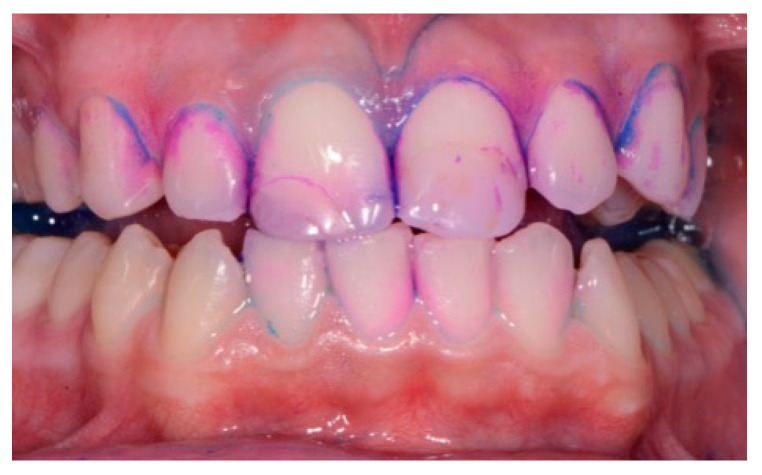
Oral biofilm detection by usage of plaque detector.

The plaque detector (GC TRI PLAQUE ID GEL) was applied with a brush on the buccal and vestibular surfaces of the teeth. The oral biofilm was detected in three different colors (Figure 5):

red/pink—indicated a recently formed bacterial biofilm;

blue/purple—indicated the presence of mature bacterial biofilm (over 48 h);

light blue/blue—accurately detected the high-risk areas where the bacteria were most active, highlighting their acidic ph.

The images were discussed with the patient and the hygienist motivated the patient regarding the importance of restoring the balance in the excessively acidic environment of the oral cavity. The D-BIOTECH clinical approach allows for minimally invasive work and acts as a guide for the operator to use these tools, which allow selective polishing and/or scaling by following the topography of the bacterial biofilm.

#### 2.1.4. Photographic Images

**Figure 6:**
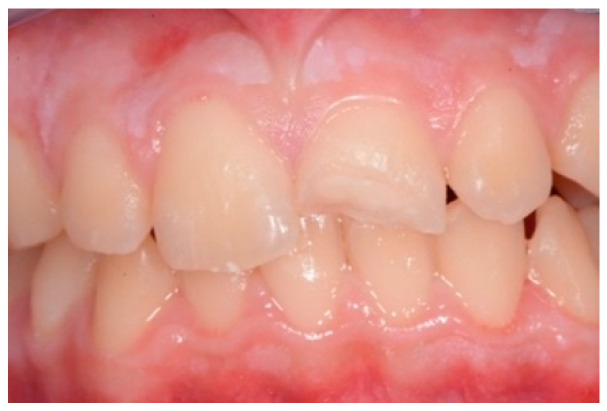
Intraoral photographic documentation.

**Figure 7:**
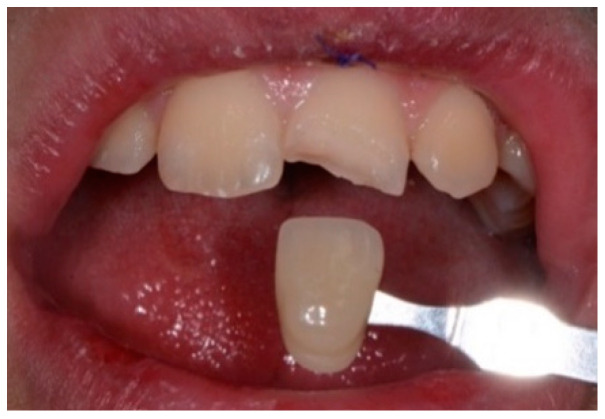
Color matching with Vita 3D Master scale.

Intraoral photographic images were collected to document the dental situation (Figure 6) and the color matching (Figure 7). Color-matching procedures are usually carried out using a Spectroshade Spectrophotometer, but in this case the Vita 3DMaster scale was preferred in order to avoid contact of the spectrophotometer with the teeth surface.

### 2.2. Treatments Performed

#### 2.2.1. Bioptron Light therapy

In the presented case, the innovation was the introduction of Polarized Polychromatic Incoherent Low Energy Radiation (PILER) using a Bioptron^®^ Device (Zepter; Wollerau, Switzerland) [17].

The Bioptron Device provides polarized visible polychromatic noncoherent light with 90 W; light wavelength = 480–3400 nm; degree of polarization = 95%; specific power density = 40 mW/cm^2^; energy density = 2.4 J/cm.

The patient started the phototherapy with the Bioptron^®^ Device (Zepter; Wollerau, Switzerland) the second day after the trauma. The applications were performed daily with a duration of 15 min at a distance of 5 cm, for 5 consecutive days, after brushing.

The subjective change of pain was measured on a visual analogue scale the day after each application, and the level of pain diminished from value 10 to value 3 after the first week of treatment, according to the following assessments:

VAS scores: day 1—10, day 2—8, day 3—7, day 4—6, day 5—5, day 6—4, day 7—3.

**Figure 8:**
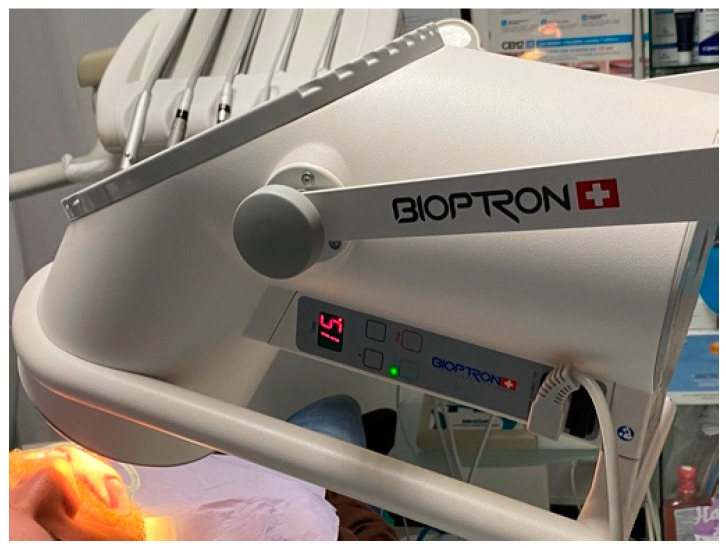
The position of the Bioptron Device and the patient’s face.

**Figure 9:**
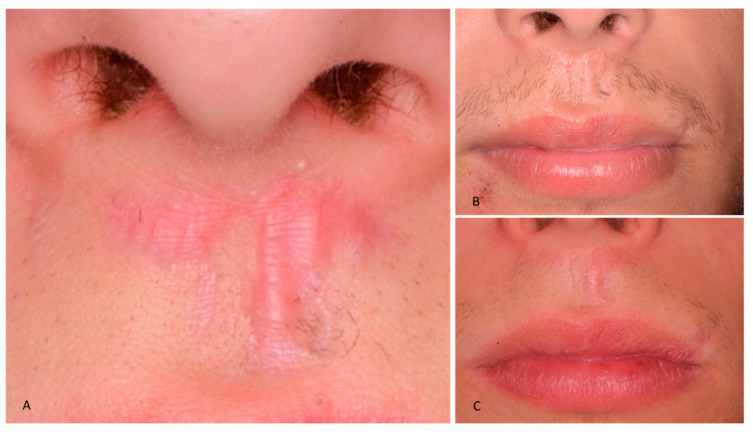
Extraoral view of the soft tissue healing after 1 month (**A**), two months (**B**) and 3 months (**C**) of phototherapy.

Then, Bioptron applications were performed during the following three months, twice per week, for a total of 24 times, 15 min duration, 5 cm distance (Figure 8); the healing of the soft tissues at three months follow-up is shown in Figure 9.

Figure 8 specifically shows the healing of the outer scar of the upper lip without any pathological changes after 24 phototherapy cycles. The lip philtrum appears to be conserved and retains its natural aesthetics and morphofunctional projection. The skin covering the scar, initially red and overgrown, has improved significantly during the first cycles with the Bioptron. The overall effect is smoothening of the tissues covering the scar and full chromatic regeneration of the skin.

On the 10th day after trauma, the plastic surgeon removed the stitches placed on the face. At this stage, it was possible to start the dental treatment.

#### 2.2.2. Tailored Brushing Method

The Tailored Brushing Method (TBM) is a novel protocol for at-home oral hygiene. This method is based on the concordance between patients and professionals to choose together the best strategies (tools and their use) for home oral hygiene procedures, regardless of the technique used [19].

The Tailored Brushing Method is based on a two-stage decision process.

FIRST STAGE

At the baseline, the following clinical characteristics of the patients are assessed:

(i) gingival biotype;
(ii) dental morphology (natural tooth, crown on natural tooth, crown on implant) and presence of diastema;
(iii) patient’s manual skills;
(iv) patient’s personality profile.

Use of the plaque test helps to communicate with the patient and to exactly show the points and areas within the oral cavity and mineralized tissue where the oral hygiene needs to be improved. Communication with the patient is achieved using new technologies, such as intra-oral cameras, that allow the collection of intra-oral images of biofilm, plaque and calculus, and the assessment of enamel hypomineralization both on vestibular and occlusal teeth surfaces.

Based on these evaluations, the clinical analysis is shared with the patient, to obtain a mutual concordance on the protocol of at-home oral hygiene to adopt. The concept of “compliance”, in which the patient passively incorporates and learns some stereotyped movements, is here innovated with the concept of “concordance”, which assumes an active interaction between the patient and the healthcare provider.

SECOND STAGE

The second stage is sub-divided into two clinical steps: Concordance1 (C1) and Concordance2 (C2).

C1 is related to the techniques of oral hygiene to be used during domiciliary oral care.

C2 is related to the choice of toothbrush and interdental brush.

TBM requires that dental hygienists and patients choose the most suitable tools to reach every dental surface, including interproximal spaces or areas commonly inaccessible with traditional techniques.

TBM recommends that toothbrushes are always intended to be used in association with interproximal brushes. Toothbrushes with an arrangement bristle in tufts disposed on multiple levels, with angled orientation, and a narrower conformation of the working part, and a handle and neck with flexibility, should be preferred. Interdental brushes made of flexible rubber are very useful, due to the ease of use and the lower risk of papillary trauma. The choice of tools is patient-tailored, based on manual skills and personality profile.

The tailored brushing protocol was used to determine and establish with the patient the personalized protocols for at-home oral hygiene procedures, immediately post-trauma and before full tooth rehabilitation.

**Figure 10:**
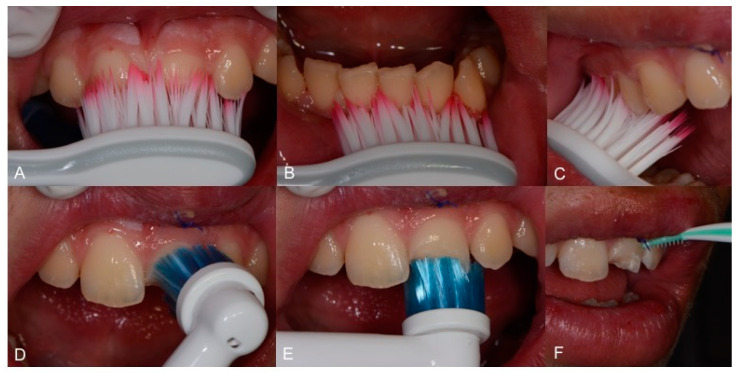
(**A**): Upper incisor brushing with manual toothbrush; (**B**): lower incisor brushing with manual toothbrush; (**C**): upper lateral brushing with manual toothbrush; (**D**,**E**): upper and lower incisor brushing with electric brush; (**F**): interdental cleaning with rubber flexile brush.

Toothbrushing instructions were given to the patient, involving use of a soft toothbrush (GUM^®^ Technique^®^ PRO toothbrush, medium bristles, Sunstar Europe, Etoy, Switzerland) and use of an electric brush. Moreover, the patient was instructed on the usage of interdental brushes. To avoid irritating the interdental papilla, interdental rubber flexible brushes (GUM Soft-Picks Advanced, Sunstar Europe, Etoy, Switzerland) were used (Figure 10).

#### 2.2.3. Oral Hygiene in Office Protocol

A professional oral hygiene treatment was performed, including airpolishing (Combi touch-all in one, Mectron, Mectron Spa, Carasco (GE), Italy) with: (i) bicarbonate powder with <120 μm to remove extrinsic stains and partially to remove calculus; (ii) glycine powder <63 μm (Mectron Glicyne powder).

**Figure 11:**
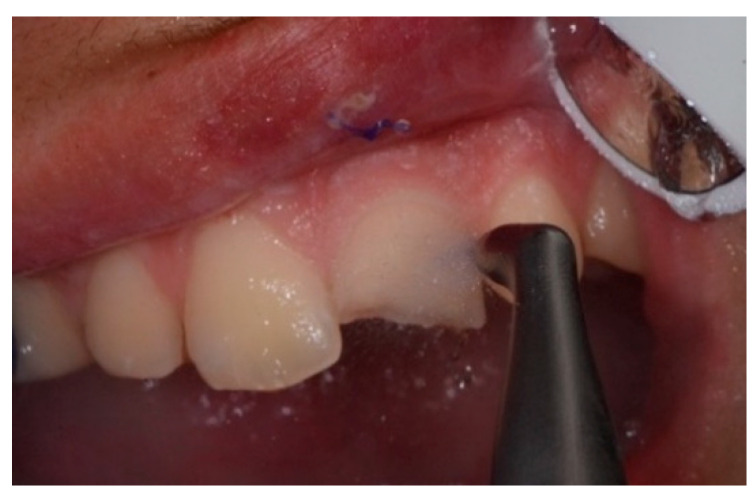
Airpolishing professional oral hygiene with bicarbonate powder.

**Figure 12:**
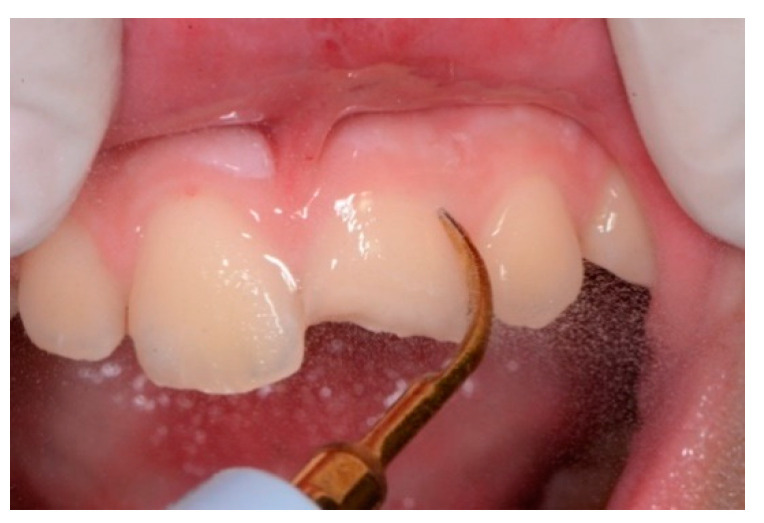
Supragingival ultrasonic scaling with a universal tip using the “soft mode” setting.

Then, supragingival ultrasonic scaling with a universal tip (Mectron S1) using the “soft mode” setting of the scaler (Mectron Multipiezo pro) was performed. The allows proper cleaning to be achieved for sensitive patients (same frequency, lower amplitude). (Figure 11 and Figure 12)

#### 2.2.4. Remineralization

Application of the remineralizing mousse (CURASEPT BIOSMALTO CARIES, ABRASION & EROSION-IMPACT ACTION MOUSSE PROFESSIONAL USE with F-ACP COMPLEX) was performed. F-ACP COMPLEX is a complex of amorphous calcium phosphate (ACP) functionalized with fluorine, carbonate and citrate. Amorphous calcium phosphate (ACP) is a highly reactive non-crystalline material that rapidly converts to hydroxyapatite. F-ACP COMPLEX is a patented technology designed to deliver, activate and bioavail this hydroxyapatite precursor [20,21]. The application of Curasept Biosmalto Professional mousse was repeated three times, at baseline, after 15 days and at two months. In addition, from the baseline and for the following 90 days, the patient applied the CURASEPT BIOSMALTO CARIES, ABRASION & EROSION-IMPACT ACTION MOUSSE HOME TREATMENT twice a day after brushing for the whole period and without interruptions.

#### 2.2.5. Desensitizing

**Figure 13:**
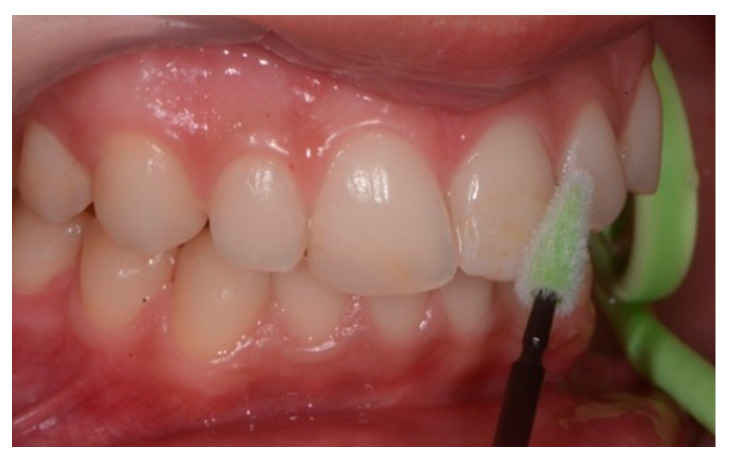
Desensitizing treatment required by the patient.

The patient reported teeth hypersensitivity when in contact with foods, liquids and air. A desensitizing treatment with application of a fluoride-based varnish (FLUOR PROTECTOR S, Ivoclar Vivadent) was performed. The varnish contains synthetic resins with insulating and protecting effects against thermal and chemical stimuli [22]. Moreover, calcium and sodium fluoride protect the enamel surface by direct remineralizing effect. (Figure 13).

#### 2.2.6. Bleaching

**Figure 14:**
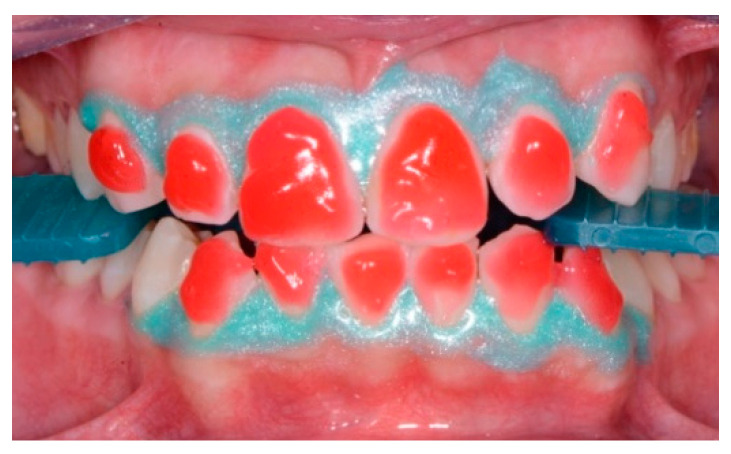
Bleaching of the upper and lower teeth with esthetic relevance.

Prior to prosthetic rehabilitation, one in-office session with 35% hydrogen peroxide bleaching was performed six months after the trauma on the upper six and lower four teeth with esthetic relevance (ENA white power, Micerium) (Figure 14).

#### 2.2.7. Rehabilitation

**Figure 15:**
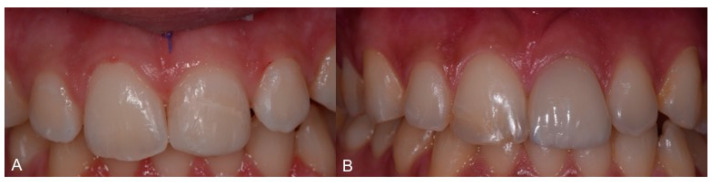
Direct restoration of 2.1 after trauma (**A**) and subsequent ceramic veneer (**B**).

A direct resin restoration was performed at the first visit and then rehabilitation with a ceramic veneer was achieved (Figure 15).

### 2.3. The recall Information

**Figure 16:**
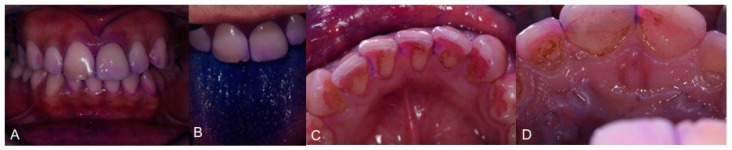
**The** TBM protocol is presented in several steps: (**A**,**B**). Plaque visualization; (**C**,**D**). Calculus visualization that allows communication with patients.

**Figure 17:**
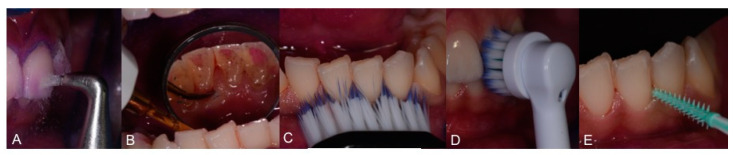
TBM protocol clinical steps: (**A**). Deplaquing; (**B**). scaling; (**C**,**D**). brushing; (**E**). interdental brushing.

During the COVID-19 pandemic it was not possible to achieve the full rehabilitation of 1.1. Figure 16 and Figure 17 show the recall procedures based on TBM, one year after trauma.

## 3. Discussion

This study presented for the first time the use of a combined approach of phototherapy and TBM to treat patients with dental trauma and concomitant soft tissue injuries. The present case report demonstrated application of a therapeutic method to manage soft and mineralized tissue trauma in an adolescent patient.

Personalized protocols in dental trauma based on TBM are shown in Figure 2.

The Tailored Brushing Method was chosen and applied based on the clinical assessment of the therapeutic needs of the patient and the specific evaluation of the gingival biotype and dental morphology. This method focused on providing the patient with the best protocols for at-home oral care. At-home care of traumatized hard and soft oral tissues can help in the overall professional management after dental trauma [1]. It has been well documented that plaque control helps in the prevention of secondary infections and the progression of root resorptive processes [22]. The Tailored Brushing Method aims to control both de-plaquing and oral biofilm removal from smooth dental surfaces and interdental spaces. Based on these goals, the use of interdental flexible rubber brushes is recommended to avoid papilla injuries caused by insufficient manual patient skills. The evaluation of this aspect is highly important, especially in a young population, where outcomes may be hampered by lack of compliance. The Tailored Brushing Method is an evolution of the well-known brushing techniques; it recognizes the importance of a dialogue between the dental professional and the patient. The choice of equipment for at-home oral hygiene is the result of this dialogue and the success of good maintenance of oral hygiene is based on the concordance between the two [19].

Data are available in the literature on brushing techniques that are based on specific movements and stereotyped timings finalized to disrupt the oral biofilm on the teeth surface [19]. Therefore, these techniques are not updated with the modern tools for oral hygiene, such as toothbrushes with easy grip handles, flexible stems, different head sizes, and rounded bristles with hardness ranging from medium to extra soft. In this clinical scenario, TBM is aimed to revise the brushing techniques and to take into account the variables that can exist in the intra-oral environment and between different personalities.

In addition, the clinical approach is innovative and based on the use of communication with patients using photos, which are taken at each visit. Images are a fundamental part of the TBM: they serve as part of the dialogue with the patient and also provide motivation. The patient is able to improve the results of treatment with a picture analysis of his progress and a detailed interview with the hygienist. Moreover, almost all of the on-board software allows collection of intra-oral images, which can be used to monitor the progression of the patient’s at-home techniques and further implementation of the tool to be used.

The TBM approach is suitable in young patients, for whom it can be difficult to achieve the needed compliance. The combined use of images and new technologies is extremely effective when a positive reinforcement of at-home oral hygiene procedures is needed. If necessary, the patient should be encouraged to modify lifestyle habits and partake in dietary counseling [23].

Phototherapy resulted in pain and stress control after trauma, with better compliance of the adolescent patient to the necessary clinical procedures. Moreover, in this case report, the application of the Bioptron Hyperlight Therapy was shown to be effective in accelerating the healing process of the injured soft tissues after trauma. It should be noted that our experience shows that the healing of urgent post-traumatic wounds, in the absence of photo-biostimulation, often evolves towards hypertrophic and/or keloid scarring, especially in young and predisposed individuals. Subsequent treatment of such pathological scars does not always reduce the aesthetic damage. These types of scar occur mainly due to the hyperactivity of fibroblasts, which leads to increased proliferation of fibrous tissue. In this sense, photo-biostimulation triggered by Bioptron Hyperlight therapy modulates the tissue response and can prevent these complications over the long term. In this specific case, healing of the outer scar of the upper lip occurred without any pathological changes, after 24 phototherapy cycles. The lip philtrum appeared to be conserved and retained its natural aesthetics and morphofunctional projection. The skin covering the scar, initially red and overgrown, improved significantly during the first cycles with Bioptron. The overall effect resulted in smoothening of the tissues covering the scar and full chromatic regeneration of the skin (Figure 8).

No data are available on Bioptron use in dental trauma. This manuscript presented for the first time the use of Bioptron polarized polychromatic noncoherent light in the treatment of a young adolescent patient after a facial trauma. The results of this case report showed that application of Bioptron soon after the trauma helped in stress and pain control, and augmented the patient’s compliance toward the necessary dental procedures. Moreover, Bioptron therapy has been shown to be effective in the healing process of the soft tissues injured after the trauma. In the present case report, the positive effect of the Bioptron on the empathy with the patient was noteworthy.

Bioptron is the standard of treatment widely used in Central and Eastern Europe. The use of such a procedure was first proposed by Hungarian scientist Mester. It is considered to be a nonaggressive, safe and cost-effective therapeutic option for the treatment of disorders of mucosa or the musculoskeletal system, or for skin eruptions [24,25,26].

Bioptron light therapy system is a device with an optical unit emitting light that is similar to the sun in terms of the electromagnetic spectrum produced, but without UV radiation. Bioptron irradiation increases metabolism in human blood cells, affecting their cytokine production and driving the immune response towards an anti-inflammatory/reparative profile [25], in addition to mitochondria activation and pain reduction [16,27,28]. These factors support the regenerative processes of irradiated tissues. However, a significant drawback of this procedure is the frequency of obligatory visits and the relatively long cumulative exposure time needed to obtain clear results. This requires significant involvement, above all, on the part of the patient. The model used in this case report is B2 device, relatively heavy (0.5 kg) [28], making it difficult to apply the procedure, which usually lasts several minutes or longer. Nevertheless, other models, smaller and lighter devices are available for home use. 

## 4. Limitations

This study represents a case report. Further research is needed in order to apply this protocol (Bioptron + TBM) to a larger patient sample size of patients with dental trauma involving soft and hard tissues. Based on the limitations stated, no definitive findings could be drawn on the use of phototherapy in dental trauma.

## 5. Conclusions

Based on the findings of this case report and on previous publications, the novel Tailored Brushing Method is successful in achieving a good patient adherence to at-home oral hygiene protocols. Bioptron light therapy was shown to be effective in the reduction of the patient’s pain and stress, and in the healing of soft tissue scars after facial and dental trauma.

## Data Availability

The data presented in this study are openly available under URL.
